# Knowledge, Awareness and Self-Care Practices of Hypertension among Cardiac Hypertensive Patients

**DOI:** 10.5539/gjhs.v8n2p9

**Published:** 2015-06-01

**Authors:** Muhammad Bilal, Abdul Haseeb, Sehan Siraj Lashkerwala, Ibrahim Zahid, Khadijah Siddiq, Muhammad Saad, Mudassir Iqbal Dar, Mohammad Hussham Arshad, Waqas Shahnawaz, Bilal Ahmed, Aimen Yaqub

**Affiliations:** 1 Dow University of Health Sciences, Karachi, Pakistan; 2 Department of Pulmonology, Jinnah Postgraduate Medical Center, Karachi, Pakistan; 3 Cardiac Surgery Department, Civil Hospital, Karachi, Pakistan; 4 Aga Khan University Hospital, Karachi, Pakistan; 5 The Lyceum, Karachi, Pakistan

**Keywords:** systolic blood pressure, hypertension, cardiac patients, outpatient department, tertiary care hospitals

## Abstract

**Introduction:**

The most prevalent form of hypertension is systolic blood pressure (SBP) and it is considered to be predisposing risk factor for cardiovascular disease. The objective of the study was to assess self-care practices, knowledge and awareness of hypertension, especially related to SBP among cardiac hypertensive patients.

**Methodology:**

A Cross sectional study was conducted on 664 cardiac hypertensive patients, which were selected by non-probability convenience sampling from cardiology outpatient department of three tertiary care hospitals. Face to face interviews were conducted using a pre designed questionnaire. Data was entered and analyzed by SPSS (V17).

**Results:**

81.8%, did not know that hypertension is defined as high blood pressure. 97.1% of the sample population did not know that top measurement of blood pressure was referred to as systolic and only 25.0% correctly recognized normal systolic blood pressure to be less than 140mmHg. 7.4% of the patients consulted their doctor for hypertension once or twice in a month. Risk factor for high blood pressure most commonly identified by the participants was too much salt intake

**Conclusions:**

The results state that there is an inadequate general knowledge of hypertension among cardiac patients and they do not recognise the significance of elevated SBP levels. There is a need to initiate programs that create community awareness regarding long term complications of uncontrolled hypertension, particularly elevated SBP levels so that there is an improvement in self-care practices of the cardiacpatients.

## 1. Introduction:

Considered as the silent killer (Hoel & Howard, 1997), high arterial Blood Pressure (BP) is the greatest causative agent for the mortality involved in cardiovascular diseases, renal failure and stroke (Rahimi&Rahimi, 2006). The studies conducted on global burden of Hypertension (HTN) reported that 25% of adults are having HTN and 9.2 % of the total deaths are because of high BP related events (Lawes, Vander Hoom, & Rodgers, 2008). As indicated by the epidemiological data, hypertension accounts for 35% of all atherosclerotic events and 49% of all cases of heart failure. It increases two to three folds of an individual’s risk for cardiovascular disease (Padwal, Strauss, & McAlister, 2001). Worldwide analysis show that the number of people with uncontrolled hypertension (>140/90 mm of Hg) rose from 600 million in 1980 to nearly 1 billion in 2008 and this number is expected to increase to 1.56 billion by 2025, which will approximately be 29% of the worldwide adult population (Sarafidis et al., 2008).

In Pakistan one in every three individual over the age of 45 years is hypertensive as revealed by the National Health Survey (NHS) (Nishtar, Faruqui, Mattu, Mohamud, & Ahmed, 2004). According to the “Sixth Report of the Joint National Committee on Prevention, Detection, Evaluation, and Treatment of High Blood Pressure”, Blood pressure is classified on the basis of Systolic blood pressure (SBP) and Diastolic blood pressure (DBP) (Lloyd-Jones, Evans, Larson, O’Donnell, & Levy, 1999). The most prevalent form of HTN is SBP and people aged above 60 years are more prone to it. Studies have shown that it is more important to control systolic blood pressure (SBP) than diastolic blood pressure (DBP) (Levine et al., 2003). Also, SBP alone is a predisposing risk factor for cardiovascular disease (CVD).Treatment and control of BP reduces the risk for cardiovascular diseases (Verdecchia, Angeli, Gentile, Mazzotta, & Reboldi, 2011). The 1990 to 1994 National Health Survey of Pakistan also showed that about 70% to 85% of Pakistani hypertensive patients were unaware of their disease, indicating limited awareness of hypertension (National Health Survey of Pakistan 1990-1994).Since there is no information available if the patients know the importance of their SBP level (Izzo, Levy, & Black, 2000), an opportunity exists to find the knowledge and awareness of the patients in controlling their SBP level.

Local data in relation to cardiac hypertensive patients is scarce, which made it necessary to conduct this study in this area to determine the association of knowledge of SBP and CVD in cardiac hypertensive population. This can help us modify the treatment options, reduce adverse outcomes and provide better health care services. The objective of the study was to assess self-care practices, knowledge and awareness of hypertension, especially related to SBP among cardiac hypertensive patients attending cardiology outpatient department (OPD) of tertiary care hospitals.

## 2. Methodology

A descriptive, cross-sectional study was conducted to evaluate the knowledge, awareness and self-care practices of hypertension among cardiac hypertensive patients. Six-sixty four patients were interviewed from the cardiology outpatient department to obtain the required information. The sample size of 664 was determined assuming percentage knowledge of HTN among cardiac patients to be 50% (taking 99% level of significance and 1% confidence limit). The blood pressure of patients was inquired by directly asking them. The study was performed after the approval from the Institutional Review Board of the Dow University of Health Sciences.

A multi-center study was conducted at the cardiology OPD of National Institute of Cardio-Vascular Diseases (NICVD), Abbasi Shaheed Hospital and Civil Hospital, Karachi. The NICVD being the largest cardiovascular tertiary care hospital of Karachi, receives patients from all over Karachi, and even from the remote areas of Sindh; while the other two, located at the two ends of the district, make it possible to cover patients from different areas of Karachi. The patients from the OPD were selected by non-probability convenience sampling, over a period of one week to prevent repetition of participants in the sample. Patients aged below 18 and pregnant women were excluded from the population, and so were the patients presenting with complications of hypertension like stroke, cerebrovascular accidents etc. and patients presenting with co morbidities like diabetes mellitus and renal failure. While the inclusion criteria included patients who were 18 and above, suffering from known pathology of valvular, ischemic heart disease or both. A patient was considered as hypertensive if he has BP > 140/90 mm of Hg or a lower BP on anti-hypertensive drugs. An interview based questionnaire was presented to each of the participant by the researcher in the homogeneous manner to limit interview biases. The first part of the questionnaire comprised of patients demographics followed by the clinical history of the patient; the second part dealt with the knowledge and awareness of hypertension with special relation to SBP, and the last part was regarding the self-care practices and management of hypertension.

The collected data was entered on SPSS (V17) software. The same software was used for data management and analysis. Mean and standard deviation were calculated for all quantitative variables like age, SBP and DBP etc. Frequency and percentages were calculated for all qualitative variables like gender etc. Chi-square test was used to find out the association benefits of study between outcome variable (knowledge of HTN) and other factors like gender, education and socio-economic status.

## 3. Results

A total of 664 cardiac hypertensive patients were included in the study consisting of 422 males and 242 females, giving a male to female ratio of 1.7:1. Table 1 depicts the demographic characteristics of cardiac hypertensive patients. Out of these 664 patients, 582 were married. The mean age of the patients was 54.4±12.5 years. Three hundred and sixty three (54.7%) of the participants were either illiterate or with level of education less than Matriculation (equivalent to grade 10). One hundred and thirty three (20.0%) of the participants were labor by profession. Consequently, monthly household income of 152 (22.9%) participants was between Rs.5000 and Rs.20000.

**Table 1:** Demographic characteristics of cardiac hypertensive patients (N = 664)

Demographic characteristics	N	%
**Age (years) Mean± SD**	54.4±12.5	-
**Gender**		
**Male**	422	63.6
**Female**	242	36.4
**Marital Status**		
**Single**	31	4.7
**Married**	582	87.7
**Divorced**	1	0.2
**Widowed**	50	7.5
**Level of education**		
**Matriculation (grade 10)**	157	23.6
**Intermediate (grade 12)**	71	10.7
**Bachelors**	54	8.1
**Masters**	19	2.9
**No education**	363	54.7
**Profession**		
**Labor**	133	20.0
**Businessman**	19	2.9
**Government employee**	39	5.9
**Private employee**	101	15.2
**Housewife**	186	28.0
**Retired**	186	28.0
**Monthly household income**		
**Less than 5000**	49	7.4
**5000-20000**	152	22.9
**20001-50000**	94	14.2
**50001-100000**	6	0.9
**Greater than 100000**	5	0.8
**Not applicable**	358	53.9

Table 2 shows the clinical history of cardiac hypertensive patients. Patients undergone angioplasty or coronary artery bypass graft surgery (CABG) were 16.3% (N=108) of the sample population and 42.8% (N=284) have been admitted in the hospital at least once in their lifetime due to uncontrolled blood pressure. Majority of the

**Table 2:** Clinical history of cardiac hypertensive patients (N = 664)

Clinical history	N	%
**Are you currently on HTN medication?**		
**Yes**	597	89.9
**No**	67	10.1
**For how long have you been suffering from HTN?**		
**< 6 Months**	149	22.4
**6 Months - 2 Years**	169	25.5
**2.1 - 5 Years**	149	22.4
**5.1 - 15 Years**	130	19.6
**> 15 Years**	67	10.0
**History of angioplasty/CABG**		
**Yes**	108	16.3
**No**	556	83.7
**Have you ever been admitted to the hospital because of uncontrolled BP?**		
**Yes**	284	42.8
**No**	380	57.2
**Family history of any disease**		
**Cardiovascular disease**	214	32.2
**Diabetes**	100	15.1
**Hyperlipidemia/Dyslipidemia**	8	1.2
**Cerebrovascular disease**	7	1.1
**Renal disease**	13	2.0
**None**	322	48.5
**Are you aware of your BP reading from your last visit?**		
**Yes**	347	52.3
**No**	317	47.7
**SBP/mm of Hg**	144.6±33.2	
**DBP/mm of Hg**	94.0±23.7	

participants, 597(89.9%), were using medications for hypertension. Most of the cardiac hypertensive patients, 169 (25.5%), said that they were hypertensive from a time period of 6 months-2 years. Out of 664 participants, 347 (52.3%) were aware of their blood pressure reading on their last visit which had a mean systolic blood pressure (SBP) of 144.6 mm Hg (SD=33.2 mm Hg) and mean diastolic blood pressure (DBP) of 94.0 mm Hg (SD=23.7 mm Hg). No family history of any disease was found in 48.5% (N=322) of the participants, however majority of the remaining participants had a family history of cardiovascular disease, (n=214, 32.2%).

Furthermore, 85.8% (N=570) of the patients claimed hypertension to be very dangerous and 89.6% (N=595) agreed that controlling blood pressure can improve a person’s health. Five hundred and forty three (81.8%) did not know that hypertension is defined as high blood pressure. Five hundred and twenty (78.3%) believed that it’s very important to take medicines to keep blood pressure under control. Majority of the sample population, 97.1% (N=645), did not know that top measurement of blood pressure was referred to as systolic and 96.8% (N=643) did not know that bottom measurement of blood pressure was referred to as diastolic. Participants who either did not know or identified normal systolic blood pressure (SBP) to be equal to 140mm Hg or greater than 140mm Hg were 50.2% of the sample population. In case of normal diastolic blood pressure (DBP), 52.6% of the participants did not know or identified the value to be equal to 90mm Hg or greater than 90mm Hg. When asked, which measure do you think is important, 70.9% were unaware. A similar outcome was seen when asked about the number responsible in causing cardiovascular disease, and 83% (N=551) of them did not know. Two hundred and thirty seven patients (35.7%) claimed to have high blood pressure at their last checkup. Results of questions relating to hypertension knowledge in cardiac hypertensive patients are given in table 3.

**Table 3:** Hypertension knowledge (N = 664)

Hypertension Knowledge	N	%
**What is the definition of HTN?**		
**High BP**	53	8.0
**High level of stress/tension**	29	4.4
**Nervous condition**	19	2.9
**High Blood Sugar**	4	0.6
**Over activity**	16	2.4
**Don’t Know**	543	81.8
**How dangerous is HTN to your health?**		
**Very dangerous**	570	85.8
**Not dangerous at all**	94	14.2
**Can lowering high BP improve a person’s health?**		
**Yes**	595	89.6
**No**	69	10.4
**What does the top number of blood pressure represent?**		
**Systolic**	19	2.9
**Diastolic**	3	0.5
**Both**	2	0.3
**Don’t know**	640	96.4
**What does the bottom number of blood pressure represent?**		
**Systolic**	4	0.6
**Diastolic**	16	2.4
**Both**	1	0.2
**Don’t know**	643	96.8
**What should be the normal top number?**		
**<140**	166	25.0
**140**	66	9.9
**>140**	153	23.0
**Don’t know**	279	42.0
**What should be the normal bottom number?**		
**<90**	315	47.4
**90**	199	30.0
**>90**	7	1.1
**Don’t know**	143	21.5
**Which measure do you think is important?**		
**Top**	54	8.1
**Bottom**	338	50.9
**Both**	133	20.0
**Don’t know**	139	21.0
**Which number is responsible in causing Cardiovascular disease?**		
**Top**	59	8.9
**Bottom**	26	3.9
**Both**	28	4.2
**Don’t know**	551	83.0
**What do you think your BP was at the last visit?**		
**High**	237	35.7
**Normal**	200	30.1
**Low**	11	1.7
**Don’t know**	216	32.5
**How important do you think medicine is to keep BP under control?**		
**Very important**	520	78.3
**Not important at all**	25	3.8
**Don’t know**	119	17.9
**Is high BP(HTN) a lifelong disease?**		
**Yes**	420	63.3
**No**	244	36.7
**Do you think high BP(HTN) is something that you can cure?**		
**Yes**	605	91.1
**No**	59	8.9

Table 4 gives the data regarding the awareness of cardiac hypertensive patients in relation to hypertension risk factors and complications. Risk factor most commonly identified by the participants was too much salt intake (N=523, 78.8%). Heart attack and stroke as a complication was recognized by 374 (56.3%) and 185 (27.9%) participants, respectively.

**Table 4:** Awareness about risk factors and complications of hypertension

Are you aware of the following risk factors of HTN?	N	%
**Too much salt intake is a risk factor of HTN**	523	78.8
**Tension is a risk factor of HTN**	354	53.3
**Lack of exercise is a risk factor of HTN**	90	13.6
**Inheritance is a risk factor of HTN**	64	9.6
**High cholesterol is a risk factor of HTN**	314	47.3
**Obesity is a risk factor of HTN**	168	25.3
**Smoking is risk factor of HTN**	155	23.3
**Aging is a risk factor of HTN**	104	15.7
**Diabetes is a risk factor of HTN**	123	18.5
**Alcohol abuse is a risk factor of HTN**	62	9.3
**Which of the following complications of HTN are you aware of?**		
**Heart attack is a complication of HTN**	374	56.3
**Stroke is a complication of HTN**	185	27.9
**Aneurysm is a complication of HTN**	20	3.0
**Narrowed blood vessels in kidney is a complication of HTN**	66	9.9
**Narrowed blood vessels in eye is a complication of HTN**	98	14.8
**Metabolic syndrome is a complication of HTN**	21	3.2
**Trouble with memory or understanding is complication of HTN**	107	16.1

Table 5 show answers to the questions related to the self-care practices of hypertension carried out by the sample population. When asked, do you regularly check your blood pressure, 459 (69.1%) claimed to check it regularly with most of them checking monthly, 234 (35.2%). Three hundred and forty three (51.7%) patients get their blood pressure checked at the nearest health care facility. Five hundred and forty six (82.2%) do not have a sphygmomanometer at home and 220 (33.1%) of them reasoned that they did not feel the need to have it. Most of the patients, 265 (39.9%), consulted doctor of hypertension every 3-6 months and 180 (27.1%) consulted their cardiologist more than every 12 months. Majority of the participants, 213 (32.1%) get their ECG examined more than every 6 months and 239 (36%) get their cholesterol checked more often than every 6 months. Taking medication was the single most important practice carried out by most of the cardiac hypertensive patients (n=329, 49.5%).

**Table 5:** Self-care practices of cardiac hypertensive patients (N= 664)

Self-care practices	N	%
**Do you have a home sphygmomanometer?**		
Yes	118	17.8
No	546	82.2
**Do you regularly check your BP?**		
Yes	459	69.1
No	205	30.9
**If yes, how often do you check your BP?**		
Daily	66	9.9
Weekly	119	17.9
Monthly	234	35.2
Every 3 months	36	5.4
More than that	4	.6
**If yes, from where do you get your BP checked?**		
At home	83	12.5
At tertiary hospital	33	5.0
Nearest health care facility	343	51.7
**What are the barriers towards self-testing for BP?**		
Expensive	168	25.3
Lack of awareness	152	22.9
Pain	6	0.9
Don’t feel the need	220	33.1
None	118	17.7
**How often do you consult your doctor for HTN?**		
Once/twice in a month	49	7.4
Every 2 months	54	8.1
Every 3-6 months	139	20.9
Every 6-12 months	265	39.9
More than 12 months	157	23.6
**How often do you consult your cardiologist for heart exam?**		
Once/twice in a month	50	7.5
Every 2 months	70	10.5
Every 3-6 months	130	19.6
Every 6-12 months	180	27.1
More than 12 months	234	35.2
**How often do you get your ECG examined?**		
Once/twice in a lifetime		
Monthly	189	28.5
Every 3 months	25	3.8
Every 6 months	118	17.8
More than that	213	32.1
Never	119	17.9
**How often do you get your blood cholesterol checked?**		
Once/twice in a lifetime	148	22.3
Monthly	122	18.4
Every 3 months	11	1.7
every 6 months	54	8.1
More than that	239	36.0
Never	90	13.6
**Which single most important practice do you carry out to control your high BP?**		
Taking medication		
Rhythmic exercise	329	49.5
Less stress	67	10.1
Quitting smoking	46	6.9
Reducing salt content in diet	48	7.2
DASH diet (diet approaches to stop HTN)	149	22.4
Losing weight	25	3.8

Further analyses were performed using demographic characteristics in which these outcomes were statistically analyzed. In general, there were no specific differences in knowledge, awareness, or self-care practices among the subgroups. Patients who had a master’s degree showed better understanding of hypertension than comparatively less educated patients. These patients specifically showed a better knowledge of the definition of hypertension (e.g., 31.6% of the participants with masters education knew that hypertension is high blood pressure compared to participants with less than master’s degree; P<0.001). In addition, businessmen tend to report better knowledge of hypertension compared to patients with other professions (e.g. 26.3% of the businessmen knew that hypertension means high blood pressure compared to patients with other professions; P<0.001).Participants with a better socioeconomic status were seen to carry out more self-care practices compared to people with low monthly household income (e.g. 76.2% of participants with monthly household income > Rs.20000 regularly checked their blood pressure compared to participants with monthly household income <Rs.20000; P<0.001).

## 4. Discussion

A descriptive study was conducted to assess the up-to-date information on knowledge, awareness and self-care practices of HTN among cardiac hypertensive patients in Karachi. We found that the awareness level of cardiac hypertensive patients in general is inadequate. The patients are not informed about recently recommended guidelines, cutoff values of SBP and association of their SBP levels with cardiovascular disease. Consequently, 48% of the patients could not recall their BP value and were unable to report whether their BP value is elevated or normal. Our results also revealed insufficient self-care practices of cardiac patients towards their BP control. However, prompt management leads to lower incidence of complications (Staessen, Wang, & Thijs, 2001) and reducing the complications of HTN is an issue of great attention (Kearney, Whelton, Reynolds, Whelton, & He, 2004).

Alarmingly, very limited numbers of cardiac patients (8%) were aware about the correct definition of HTN and majority of the patients (82%) did not know anything about this term. Less than 3% could identify the top and bottom number of BP reading as systolic and diastolic correctly. This reveals poor awareness level of the participants. This is fairly consistent with the findings of another Pakistani study which has also narrated inadequate awareness among hypertensive patients (Zafar, Gowani, Irani, & Ishaq, 2008). However, this is incompatible with the international studies conducted in China (Zhang et al., 2009) and USA (Ong & Cheung, 2007) which have reported improved awareness and knowledge. These differences in results could be attributed to significant difference in literacy rates as more than half of the participants of our study had no education.

It was reassuring to see that majority of the patients were knowledgeable about the seriousness of the condition to their health due to uncontrolled BP. Ninety percent of the patients knew that lowering BP would improve a person’s health and that they could lower their uncontrolled BP by adjusting their lifestyle. The results are in line with the international study conducted by Susan et al. (Oliveria, Chen, McCarthy, Davis, & Hill, 2005). This shows their willingness to improve their blood pressure, but due to inadequate knowledge they are unable to do so.

During recent years, SBP has been recognized as the greatest causative agent for the morbidity and mortality associated with CVD and stroke (Oliveria, Chen, McCarthy, Davis, & Hill, 2005). In several clinical trials it has been demonstrated that a greater reduction in cardiovascular events occur with the treatment of elevated SBP level (Staessen et al., 1997). The results of our study suggest that majority of our patients were unaware of the importance of SBP with respect to management of their heart disease. When they were questioned about which measure is more important, 51% reported DBP and only 8% answered SBP. Sixty-five percent of the patients did not know the normal values of SBP or wrongly reported it as 140 mm Hg or greater. However, 77% of the participants correctly quoted normal DBP as 90 mm Hg or less, which suggests that they are aware of the cut point for DBP. Only very few people knew that top number is responsible for CVD. These findings are similar to those of a study conducted on patients of Henry Ford Health System in Michigan (Oliveria, Chen, McCarthy, Davis, & Hill, 2005). The results indicate that physicians must emphasize their patients on importance of elevated SBP and cardiovascular risk.

A large number of participants considered salt intake as the risk factor to be associated with high BP, which is evident from a study done on British population (Ashfaq, Anjum, Siddiqui, Shaikh, & Vohra, 2007) and in line with findings of another Pakistani study (Beard, Blizzard, O’Brien, & Dwyer, 1997). Poor awareness was associated with smoking, diabetes mellitus and alcohol intake which is further consistent with the study conducted on hypertensive patients of Pakistan (Zafar, Gowani, Irani, & Ishaq, 2008). The local data has reported an increase in alcohol consumption in Pakistan and regularity of smoking among adults (Ali, Sathiakumar, & Delzell, 2006) and children (Rozi, Akhtar, Ali, & Khan, 2005). As a result, people must be emphasized with the risk factors linked with diabetes mellitus, smoking and excess alcohol intake. A significant number of patients were aware with the fact that stroke and CVD are complications of uncontrolled BP which is similar to findings of Line Aubert (Aubert et al., 1998), Susan (Oliveria, Chen, Mccarthy, Davis, & Hill, 2005), and a Pakistani study (Ashfaq, Anjum, Siddiqui, Shaikh, & Vohra, 2007). The results could be overestimated as the study was conducted in cardiology OPD. Conversely, most of the patients were not aware about complications related to eye, kidney, metabolic syndrome and trouble with memory which is consistent with results of other researchers (Kearney et al., 2005). Prompt education of hypertensive patients on complications is essential to increase their drug compliance. An international survey has revealed better drug compliance and follow-up visit among patients who were aware with the morbidities and reduction in life expectancy associated with high BP (Balazovjech & Hnilica, 1993).

Furthermore, patient’s regularity on visiting doctor for HTN and cardiologist for their cardiovascular exam, including ECG and cholesterol level, and having home sphygmomanometer was found to be very low. The results could be attributed to less affordability as most of the patients belonged to low socio-economic status. The attitude of patients to bring about lifestyle changes like rhythmic exercise, weight reduction and less stress was significantly less. This might be because of the perception that lifestyle changes have no impact on high BP management as shown by Aubert (Kearney, Whelton, Reynolds, Whelton, & He, 2004). According to the study, DASH diet and reduced salt intake improves BP control (Zafar, Gowani, Irani, & Ishaq, 2008) but participants of our study have shown extremely less compliance to these approaches. These results further evaluate insufficient self-care practices of HTN among cardiac patients.

To the best of our knowledge, our research has comprehensively evaluated HTN knowledge especially related to SBP among cardiac patients along with their self-care practices involved in the management of high BP. However, there were a few limitations to our study. Most of the participants belonged to low socioeconomic status and had no education. So the results cannot be generalized to the entire population. Secondly, our sample does not include those who were unstable to attend cardiology OPD. Their views may vary over knowledge and self-care practices of HTN. Apart from this we did not obtain the BP values from medical records for the participants who could not recall their last BP reading.

## 5. Conclusion

According to our results, there is an inadequate general knowledge of hypertension in cardiac patients. Patients do not recognize the significance of elevated SBP levels. The study helped us identify the areas that need to be considered by the awareness programs. People should be encouraged to have their blood pressures checked regularly, especially when they have a family history of hypertension. There is a need to initiate programs that create community awareness regarding long-term complications of uncontrolled hypertension in cardiac patients, particularly elevated SBP levels. Improvement in self-care practices can decrease the mortality rate associated with hypertension.
